# The Prevalence of Body Dysmorphic Disorder and Its Associated Risk Factors Among Dental Patients: Why Are My Patients Not Satisfied?

**DOI:** 10.7759/cureus.49739

**Published:** 2023-11-30

**Authors:** Abdulrahman Alharbi, Ali Alkhathami, Faraz A Farooqi, Khalifa S Al-Khalifa, Suliman Shahin, Essam Nassar, Balgis Gaffar

**Affiliations:** 1 College of Dentistry, Imam Abdulrahman Bin Faisal University, Dammam, SAU; 2 College of Dentistry, Department of Dental Education, Imam Abdulrahman Bin Faisal University, Dammam, SAU; 3 College of Dentistry, Department of Preventive Dental Sciences, Imam Abdulrahman Bin Faisal University, Dammam, SAU

**Keywords:** dysmorphophobia, dysmorphia, mental health, dental aesthetics, body dysmorphic disorder

## Abstract

Background: Body dysmorphic disorder (BDD) is a common disorder that consists of a distressing or impairing preoccupation with imagined or slight defects in appearance. In dentistry, those patients will have unrealistic expectations and usually will be unsatisfied with the outcomes of dental treatment. This study investigated the prevalence of BDD and its associated factors among dental patients.

Material and methods: In this cross-sectional survey-based study, a purposive sample was used to recruit adult patients seeking dental treatment in private and public facilities. Data was collected using the validated Arabic version of the Cosmetic Procedure Screening Questionnaire (COPS) for Body Dysmorphic Disorder, a validated nine-item self-administrated instrument that is scored from zero (least impaired) to five (most impaired). Chi-Square, Mann-Whitney U, and Fisher’s Exact tests were used to compare the associations between the study variables and BDD.

Results: A total of 507 patients responded to the questionnaire. The prevalence of BDD was 34.1%. The average age of the participants was 35.2 years; the majority were males 290 (57%) and Saudis 436 (86%) with a college education 304 (60%). None of the demographics was associated with BDD but the type of treatment was significantly associated with BDD with teeth crowning and restorations.

Conclusion: This study provides insights into the prevalence of BDD and its associated factors among dental patients in Eastern Saudi Arabia. The findings highlight the need for further research to better understand the factors contributing to the development of BDD and to guide prevention and intervention strategies in dental settings.

## Introduction

Body dysmorphic disorder (BDD) is a common disorder that consists of a distressing or impairing preoccupation with imagined or slight defects in appearance [1]. Formerly known as dysmorphophobia, BDD is also known as dysmorphia or dysmorphic syndrome, it was first described as dysmorphophobia [2]. Normative discontent [3] or dysmorphic concern [4] is a phenomenon where an individual is preoccupied with their physical appearance, but it does not cause significant distress or impairment. The causes of BDD are not fully understood but are likely influenced by a combination of biological, psychological, and cultural factors [5]. It often starts in adolescence and can persist over time, with individuals becoming preoccupied with multiple body parts [6]. The American Psychiatric Association designated BDD as a compulsive disorder because there is a pathological fear of ugliness with regard to some parts of the body even though no defect is observed by others or, if observed, is thought to be minor [7]. It is associated with marked impairment in psychosocial functioning, poor quality of life, and high suicidal rates [1]. Acne, wrinkles, scars, vascular markings, swelling, facial asymmetry or disproportion, hair thinning, and excessive facial hair appear to be the most concerning issues among BDD patients [8]. Patients suffering from BDD are also concerned about their noses, eyes, eyelids, brows, ears, cheeks, teeth, lips, mouth, and jaws [9].

The most reported reasons for BDD patients to seek dental treatment were orthodontic treatment and teeth whitening [10]. Rotations, spacing, upper and lower midlines, and tooth size were some of the orthodontic concerns [11]. The incidence of BDD has increased among patients seeking aesthetic treatment; however, data evaluating BDD in the dental field are limited. Rodríguez et al. found that the prevalence of BDD in general dental practice and prosthodontics was 7% [12]. In 2006, Hepburn and Cunningham surveyed 40 patients attending orthodontic treatment and found an estimated BDD prevalence of 7.5% [13]. In 2008, the majority of patients in different dental specialties such as orthodontics, prosthetics, and restorative dentistry were found to present with aesthetic problems [14]. In another study done in 2014, a total of 270 orthodontic patients were evaluated for BDD, and 15 patients (5.5%) were positive for BDD [15]. In 2020, a study was conducted on 1184 patients with varying degrees of malocclusion, they found that the prevalence of BDD among orthodontic patients was 5.2% [16]. Furthermore, BDD appears to pose a public health concern as suicidal rates among individuals identified with BDD have shown to be high [17].

Due to the paucity of studies in dentistry and the lack of awareness of BDD among dentists, the prevalence of BDD is not recognized and effectively treated. Identification of BDD in dental practice is important as those patients may have unrealistic expectations and they are usually unsatisfied about the outcome of dental treatments. Patients’ satisfaction is an important element in successful dental services [10]. It is therefore essential to understand all aspects that can affect patients’ satisfaction. Given the influence of factors such as clinical settings, personal characteristics, and behaviors on BDD there is a need for reports from different dental specialties to have a better understanding of BDD in general. Therefore, this study aimed to investigate the prevalence of body dysmorphic disorder and its risk-associated factors among dental patients in Eastern Province, Saudi Arabia.

## Materials and methods

Study design and setting

This cross-sectional survey-based study was conducted in selected public and private dental hospitals/clinics in the Eastern province of Saudi Arabia during the period from October 2022 to April 2023.

Study participants

The study included adult patients seeking dental treatment in the Eastern province of Saudi Arabia. Inclusion criteria were patients seeking dental treatment in different specialties (orthodontics, prosthodontics, endodontics, periodontics, and restorative). Pediatric patients, patients seeking emergency treatment patients with physical deformities, patients taking anti-psychotic drugs, craniofacial syndromes, cleft lip and/or palate, and skeletal malocclusion requiring orthognathic surgery were excluded from the study.

Sample size and sampling technique

Based on a sample size calculation with the following criteria, there was a 5% margin of error, a 0.05 alpha level, and a 5% expected BDD prevalence, the sample size needed was 377, which was increased to 500 to compensate for any missing information or incomplete data. The sample size was calculated using Raosoft [18] an electronic sample size calculator.

Data collection tool

Data was collected using the validated Arabic version of the Cosmetic Procedure Screening Questionnaire (COPS) for BDD [19,20]. The COPS is a brief (5 minutes to complete) self-report using nine questions that are scored from one (least impaired) to five (most impaired). It evaluates current or lifetime BDD according to certain criteria. In addition, it evaluates whether the participants are concerned about their appearance and, if they are, whether this impairs their functionality.

The score was achieved by summing the nine items where item numbers 2, 3, and 5 were reversed scored. A score of 24.75 or above is strongly suggestive of a diagnosis of BDD. 

Data collection procedure

The data for this study was collected using an online questionnaire that was distributed to a sample of participants recruited through various channels including social media platforms, and in person in private clinics, and public hospitals. The questionnaire was made available through Google Forms, and participants were directed to it through a QR barcode posted on the clinics and via WhatsApp links sent to recruited individuals.

Ethical considerations

This study was approved by the Deanship of Scientific Research-Imam Abdulrahman Bin Faisal University (IRB-2022-02-373). The questionnaire was preceded by an introduction explaining the study, its purpose, and the time to complete the survey. Participants were assured of the confidentiality of their information, that no personal information will be collected, that they could quit the survey at any time, and that once they choose to submit their responses cannot be retrieved. Choosing to proceed with the survey and submit was considered as consent.

Statistical analysis

The data was analyzed using IBM SPSS Statistics for Windows, Version 28 (Released 2021; IBM Corp., Armonk, New York, United States). Mean, standard deviation, frequency, and percentages were calculated as descriptive statistics where appropriate. For measuring the association between demographic information, satisfaction with the last dental treatment, and BDD status, a Chi-square test was used. The odds of having BDD in comparison to demographic information are calculated with logistic regression and presented with a 95% confidence interval in a table. The score of the BDD scale was compared using the Mann-Whitney U test. A p-value ≤ 0.05 was considered statistically significant.

## Results

Out of 507 total patients who were included in the final analysis, 173 (34.1%) were diagnosed with BDD which shows that the prevalence of BDD was 34.1% among the studied samples. The average age of the participants was recorded as 35.21+12.8 years old with the range of 18 to 85 years with a higher portion of males 290 (57%) compared to females. Most of the participants are Saudis 436 (86%) and have college education 304 (60%).

Depending on the total score, BDD participants were categorized as those who have BDD and those who did not have BDD and Table 1 shows the comparison of the prevalence of BDD with demographical characteristics. From the total, 103 (35.5%) males were diagnosed with BDD and 70 (32.3%) females; however, there was no significant association between gender and the prevalence of BDD (p=0.251). Participants in the BDD group were marginally older (36.69+12.43 years) compared to those with no BDD (34.79+12.94) but the difference was not statistically significant (p=0.981). BDD presence was observed in 108 (35%) married participants and 65 (33%) single participants with no significant association (p=0.396).

**Table 1:** Demographic data of the study participants in association with BDD. BDD: Body dysmorphic disorder

Demographical variables		BDD categories		p-value
		Normal	BDD	
		N=334	N=173	
		Mean	SD	
Age		34.79+12.94 years	36.69+12.43 year	0.98
		N(%)	N(%)	
Gender	Male	187 (64.5)	103 (35.5)	0.251
	Female	147 (67.7)	70 (32.3)	
Nationality	Saudi	290 (66.5)	146 (33.5)	0.268
	Non-Saudi	44 (62)	27 (38)	
Education	School	124 (66)	64 (34)	0.528
	College/Diploma	210 (65.8)	109 (34.2)	
Social Status	Married	203 (65.3)	108 (34.7)	0.396
	Single	131 (66.8)	65 (33.2)	
Did you have plastic surgery	Yes	300 (66.8)	149 (33.2)	0.534
	No	34 (58.6)	24 (41.4)	

Table 2 compares the types of last dental clinic visits between the BDD and non-BDD groups. The most significant reason for visiting the dental clinic among the BDD participants was for teeth restorations 96 (55.5%) (p=0.028) and dental crowns 83 (48%) (p=0.001) followed by extraction 69 (39.9%). Esthetic surgery was the least choice among BDD participants for visiting the dental clinic 9 (5.2%).

**Table 2:** Type of dental treatment as reported by the study participants in association with BDD. * statistically significant; **highly significant; BDD: body dysmorphic disorder

Type of Treatment	BDD Categories		
	Normal n (%)	BDD n (%)	p-values**
Emergency treatment	89 (26.6)	46 (26.6)	0.539
Teeth braces	53 (15.9)	26 (15)	0.457
Teeth crowns	88 (26.3)	83 (48)	0.001**
Teeth restorations	216 (64.7)	96 (55.5)	0.028*
Esthetic surgery	15 (4.5)	9 (5.2)	0.437
Gingival treatment	57 (17.1)	34 (19.7)	0.273
Extraction	132 (39.5)	69 (39.9)	0.506

Table 3 compares the satisfaction with the most recent dental treatment received by participants with BDD and those without BDD status. Among the satisfied participants, most of them belonged to the BDD group 28 (62.2%) vs 17 (37.8); however among the not-satisfied participants, most of them belonged to the normal group (69% vs 30.8%), and the association between treatment satisfaction with the prevalence of BDD was statistically significant (p<0.05).

**Table 3:** Satisfaction with last dental treatment in association with BDD. **Highly significant; BDD: body dysmorphic disorder

Satisfaction with last Dental Treatment	BDD Categories		p-value
	Normal n (%)	BDD n (%)	
Extremely satisfied	17 (37.8)	28 (62.2)	0.001**
Satisfied	73 (64.6)	40 (35.4)	
Acceptable	89 (71.2)	36 (28.8)	
Not acceptable	155 (69.2)	69 (30.8)	

Table 4 presents the BDD item comparison with the gender and education level of the participants. Average scores were significantly higher in female participants for items # 1 (4.22±0.784) and 4 (4.76±0.758) which were reverse-coded for analysis as well. However, in BDD item numbers 6, 7, and 8, the male participant’s score was significantly higher (1.66±1.154, 1.69±1.117, and 1.59±1.082 respectively). On the other hand, in the participants with higher degrees the average score was found higher in item numbers 1, 2, and 4 which were related to the avoidance of activities, ugly teeth, and frequency of checking teeth (4.7±0.871, 4.04±1.146, 4.35±0.689 respectively), whereas the score was higher in the school degree holder regarding teeth interference with the ability to work or study (1.61±1.096 vs 1.41±0.947, 0.009).

**Table 4:** BDD item comparison with gender and education level of the participants *Statistically significant; BDD: body dysmorphic disorder; SD: standard deviation.

BDD Items	Gender			Education			Total Score Mean (SD)
	Male Mean (SD)	Female Mean (SD)	P-value	School Mean (SD)	College/Diploma Mean (SD)	P-value	
How often do you deliberately check your teeth? Not accidentally catch sight of it. Please include looking at your feature in a mirror or other reflective surfaces like a shop window or looking at it directly or feeling it with your fingers	4.37±0.71	4.22±0.784	0.047*	4.22±0.828	4.35±0.689	0.023*	4.3±0.746
How much do you feel your teeth is currently ugly, unattractive or not right?	3.86±1.236	4.16±1.077	0.201	3.9±1.23	4.04±1.146	0.005*	3.99±1.179
How much does your teeth currently cause you a lot of distress?	2.07±1.236	2±1.18	0.182	2.13±1.27	1.98±1.175	0.576	2.04±1.212
How often does your teeth currently lead you to avoid situations or activities	4.44±1.196	4.76±0.758	0.001*	4.38±1.259	4.7±0.871	0.001*	4.58±1.042
How much does your teeth currently preoccupy you? That is, you think about it a lot and it is hard to stop thinking about it	1.95±1.199	1.94±1.104	0.177	2.04±1.242	1.89±1.105	0.909	1.95±1.158
If you have a partner, how much does your teeth currently have an effect on your relationship with an existing partner? If you do not have a partner, how much does it have an effect on dating or developing a relationship?	1.66±1.154	1.55±1.013	0.034*	1.75±1.244	1.54±0.993	0.268	1.62±1.096
How much does your teeth currently interfere with your social life	1.69±1.117	1.5±1.01	0.008*	1.77±1.195	1.51±0.987	0.057	1.61±1.075
How much does your teeth currently interfere with your ability to work or study, or your role as a homemaker? (Please rate this even if you are not working or studying: we are interested in your ability to work or study.)	1.59±1.082	1.35±0.886	0.03*	1.61±1.096	1.41±0.947	0.009*	1.49±1.009
How much do you feel your appearance is the most important aspect of who you are?	2.6±1.32	2.78±1.399	0.116	2.55±1.392	2.75±1.332	0.125	2.68±1.356

## Discussion

This study aimed to investigate the prevalence of BDD among a sample of dental patients in Eastern Saudi Arabia using a validated questionnaire. The results showed a majority of participants expressed contentment with their physical appearance. Among the patients who screened positive for BDD, a significant correlation was found between satisfaction with the last dental treatments and the presence of BDD. None of the demographics were associated with increased risk of BDD, however gender differences existed per item score. This study provides insights into the prevalence of BDD among dental patients seeking dental treatment in Saudi Arabia.

In the current study, we found the prevalence of BDD to be 22.3% higher than what was reported from studies conducted among orthodontic patients in other cultures [15,16,21]. Cross-cultural BDD differences have been previously demonstrated by Bohne et al. [22]. This increased prevalence might be explained by the increased aesthetic awareness of patients seeking dental treatment nowadays or social pressures posed on society stemming from increased exposure to aesthetic demanding services among the modern populace. Many demographic and personal factors are thought to influence individuals’ satisfaction with body image and self-perception of one’s looks [23,24]. In the current study, we failed to establish an association between BDD and gender contrary to the findings of Veale et al., 2016 who found that BDD was more prevalent among females [25]. The same observation was also reported by Rodríguez et al., who found that women were more likely than men to have BDD [12]. However, it is important to note that our study may have been limited by factors such as the sample size and the potential cultural influences, which could have acted as possible confounders [21,22].

We found that those with BDD had a slightly higher mean age; however, this difference was also not statistically significant. The educational level was one of the personal characteristics that was reported to influence body image [23], but no association was established in the current study. Similarly, although the percentage of married participants in the BDD group was higher compared to singles, this difference was also not statistically significant. In this study, it was found that there was no significant association between nationality and the presence of BDD with the majority of the study samples being Saudi nationals. A recent study using the “Yale-Brown Obsessive-Compulsive Scale modified for Body Dysmorphic Disorder questionnaire” also reported no association between any of the demographics and BDD [26]. The effect of culture has been studied on many constructs that reflect perceptions of body image [23] such as facial, body appearance [27], and body weight [28]. A recent study conducted in another city in Saudi Arabia came out with the same lack of association between any of the demographics and BDD [29]. As such further research is needed to explore the underlying factors contributing to the diminished effect of demographics on BDD, to determine if it is specific to the Saudi population or applicable to other populations as well.

Among BDD participants, the most common reasons for visiting the dental clinic were teeth crowning and teeth restoration, both accounting for more than third of the visits. This aligns with previous research that has demonstrated a higher prevalence of BDD among individuals seeking esthetic dental procedures. Previous studies have demonstrated that patients seeking orthodontic treatment are associated with BDD [15,16]. However, our study population did not find such an association. For instance, a study reported that one-fifth of female patients seeking cosmetic procedures were diagnosed with BDD [27]. These findings suggest that individuals dissatisfied with their appearance may be more inclined to seek dental restorations and other esthetic procedures [29]. Similar to the work by Grant et al. 2002 [30] and Cerea et al. 2022 [31] which have shown a relationship between BDD symptoms and "not just right" experiences (NJREs) in individuals seeking cosmetic procedures. NJREs refer to feelings of dissatisfaction or discomfort with one's appearance, which are some characteristics of BDD. The presence of NJREs has been identified as a vulnerability factor for BDD symptoms in the context of plastic surgeries and aesthetic medical settings [30,31]. This suggests that individuals seeking cosmetic dental procedures, such as teeth crowning and teeth restoration, may be more likely to have BDD symptoms and experience NJREs. In addition to our findings, other studies have also explored the relationship between BDD and cosmetic procedures. For example, a study by Raeissosadati et al. in 2022 [32] compared the frequency of BDD in women undergoing abdominoplasty with those attending other plastic surgery clinics [32]. They found a significant difference in the BDD score between the abdominoplasty group and the other group, indicating a higher prevalence of BDD among abdominoplasty applicants [32]. Overall, our findings are consistent with previous research that has shown a high prevalence of BDD among individuals seeking cosmetic procedures [27]. Suggesting that individuals who are dissatisfied with their appearance may be more likely to seek dental restorations and other cosmetic dental procedures. These findings highlight the importance of addressing self-esteem and core beliefs in the prevention and treatment of BDD [33,34].

The findings of this study also highlight the significant impact of BDD on satisfaction levels with dental treatments. Individuals with BDD may have increased sensitivity to perceived flaws in their appearance, leading to increased dissatisfaction with dental procedures. Dental professionals should be aware of the potential influence of BDD on patients’ satisfaction and tailor their approach accordingly. Additional research is also needed to explore interventions and strategies that can enhance the satisfaction levels of individuals with BDD undergoing dental treatments.

Limitations

It is important to acknowledge the limitations of our study, including the potential for selection bias and the need for the replication of the study in a larger, more diverse sample. Additionally, the cross-sectional nature of our study limits our ability to establish causality or determine the temporal relationship between BDD and the identified factors. Social media use is one of the major factors listed by many researchers [34] as a drive to seek cosmetic treatments which was not investigated in the current study. Further investigation is warranted to gain a deeper understanding of the association between BDD and cosmetic procedures, as well as to develop effective psychological interventions for individuals with BDD who seek cosmetic dental treatments. It is important to note that aesthetic dental work performed in general practice encompasses treatments that are both clinically necessary and those that are undertaken when no underlying disease process exists. These two distinct patient groups may exhibit varying levels of baseline satisfaction, which should be further examined through additional research. Further, future research might be aimed at establishing links of BDD with quality of life.

## Conclusions

The prevalence of BDD among the surveyed dental patients was comparably high. Teeth crowning and restoration were the most common reasons for visiting the dental clinic among those with BDD. None of the demographic characteristics such as gender were associated with BDD. The study also emphasized the significant impact of BDD on satisfaction levels with the last dental treatment, suggesting the need for tailored approaches by dental professionals.
